# Uridine Ameliorates Dextran Sulfate Sodium (DSS)-Induced Colitis in Mice

**DOI:** 10.1038/s41598-017-04041-9

**Published:** 2017-06-20

**Authors:** Manish Kumar Jeengar, Dinesh Thummuri, Mattias Magnusson, V. G. M. Naidu, Srinivas Uppugunduri

**Affiliations:** 1 Autoimmunity & Immune Regulation (AIR), Department of Clinical & Experimental Medicine, Linköping University, Linköping, Sweden; 2 Department of Pharmacology & Toxicology, National Institute of Pharmaceutical Education & Research Institute, Balanagar, Hyderabad 500037 India; 3 Department of Pharmacology & Toxicology, National Institute of Pharmaceutical Education & Research Institute, Guwahati, 781032 Assam India; 4 Regional Cancer Center South East Sweden and Department of Clinical and Experimental Medicine, Linköping University, Linköping, Sweden

## Abstract

Uridine, one of the four components that comprise RNA, has attracted attention as a novel therapeutic modulator of inflammation. However, very little is known about its effect on intestinal inflammation. The aim of the present study was to investigate the potential protective effect of intracolonic administered uridine against DSS induced colitis in male C57BL/6 mice. Intracolonic instillation of 3 doses of uridine 1 mg/Kg (lower dose), 5 mg/Kg (medium dose), and 10 mg/Kg (higher dose) in saline was performed daily. Uridine at medium and high dose significantly reduced the severity of colitis (DAI score) and alleviated the macroscopic and microscopic signs of the disease. The levels of proinflammatory cytokines IL-6, IL-1β and TNF in serum as well as mRNA expression in colon were significantly reduced in the uridine treated groups. Moreover, colon tissue myloperoxidase activities, protein expression of IL-6, TNF- α, COX-2, P-NFkB and P-Ikk-βα in the colon tissues were significantly reduced in medium and high dose groups. These findings demonstrated that local administration of uridine alleviated experimental colitis in male C57BL/6 mice accompanied by the inhibition of neutrophil infiltration and NF-κB signaling. Thus, Uridine may be a promising candidate for future use in the treatment of inflammatory bowel disease.

## Introduction

Inflammatory Bowel Disease (IBD) is a broad term referring to conditions with chronic inflammation of the gastrointestinal tract. Ulcerative colitis (UC) and Crohn’s disease are two common forms of IBD that share some characteristics, but also exhibit distinct differences in risk factors, genetic predisposition and clinical and histological features^1^.

Crohn’s disease can affect the entire gastrointestinal tract and presents frequently with abdominal pain, fever, and clinical signs of bowel obstruction or diarrhoea with passage of blood and mucus. It has been postulated that the pathophysiology Crohn’s disease is mediated and perpetuated by an imbalance of effector Th-1 or Th17 cells responsible for secretion of interferon RecNNFα, and interleukins 17 and 22, versus naturally regulatory T cells responsible for secretion of interleukin 10 and transforming growth factor [TGF]^2^. Rapid influx and retention of leukocytes, a known feature of Crohn’s disease, is mediated by chemokines, selectins, integrins and their respective ligands (immunoglobulin superfamily, ICAM-1, MAdCam-1)^3, 4^.

Inflammation in UC occurs typically in the colon and rectum^5^. Symptoms include the development of bloody diarrhoea with or without mucus, rectal urgency, tenesmus, abdominal pain, weight loss, fatigue and extraintestinal manifestations^6^. It has been suggested that ulcerative colitis is associated with an atypical Th2 response mediated by non-classic natural killer T-cells producing interleukins 5 and 13, the latter being highly cytotoxic to epithelial cells which further increases intestinal permeability^7^. Tumor necrosis factor −α (TNF), which is elevated in the blood, stool samples and mucosa of patients with ulcerative colitis, also induces apoptosis in intestinal epithelium and is an important and effective target for controlling the disease^8^. E and P-selectin are up-regulated on the vascular endothelium of ileum and colon and control migration of leukocytes especially lymphocytes to the intestinal lamina propria^9^. Further, the mucosal vascular addressin cell adhesion molecule (MAdCAM-1), expressed on HEV’s of the intestinal lamina propria, binds to α4β7 integrin and controls T-cell traffic to the gut-associated lymphoid tissue^10^.

It is now well documented that commensal bacteria are involved in the pathophysiology and disease progression of IBD^11^. The trigger for the initial leakage is unknown, but, once the epithelial barrier is dysfunctional, bacterial components activate production of cytokines like TNF, IL-6, IL-1β^9, 12^. This leads to further recruitment of neutrophils and macrophages that are more susceptible to bacterial stimulation than the resident macrophages and epithelial cells, on which the bacterial recognition receptors such as TLR or CD14 are down regulated^8, 13^. Modern treatments of IBD, including suppression of inflammation using 5-aminosalicylic acid, corticosteroids, immunomodulators and biological agents have proven to be efficacious^2^. However, these therapies are associated with major adverse effects^14^. Since none of the existing therapeutic modalities are able to provide complete or long lasting disease remission, there is still a pressing need to find better therapies for inflammatory bowel disease.

The DSS induced colitis model is a relevant model for translation of mice data to human disease and the model has been validated by using different therapeutic agents for human IBD^15^. Several studies have demonstrated that DSS induces breakdown of the mucosal epithelial barrier, allowing entry of luminal microorganisms into the mucosa, resulting in an overwhelming inflammatory response including NF-κB activation, over expression of pro-inflammatory cytokines and clinical symptoms of colitis^16–18^.

Uridine, a small and inexpensive pyrimidine nucleoside, is essential for synthesis of RNA and biomembranes. Uridine has attracted attention due to its anti-inflammatory effect in a rabbit dry eye model and an animal model of asthma^19, 20^. We have previously shown that uridine inhibits leukocyte adhesion *in vitro* and is a potent inhibitor of leukocyte extravasation in a model of sephadex induced lung inflammation^21^. Also, local administration of uridine into knee joints was able to protect against antigen induced arthritis in mice^22^. Although the exact mechanism of action of uridine is still unclear, it has been hypothesised that uridine could inhibit selectin-mediated adhesion through direct binding to selectins^23^. Another mechanism of action could be through generation of uridine-5′diphosphate (UDP) and uridine-5′-triphosphate (UTP) that can bind and activate the P2Y_2_, P2Y_4_ and P2Y_6_ receptors^20^.

Since, IBD including UC and Crohn’s disease are also chronic inflammatory diseases, we hypothesized that uridine could be effective in the treatment of IBD. Hence, the present study was undertaken to investigate the anti-inflammatory potential of locally administered uridine in DSS induced colitis in mice.

## Results

**Figure 1:**
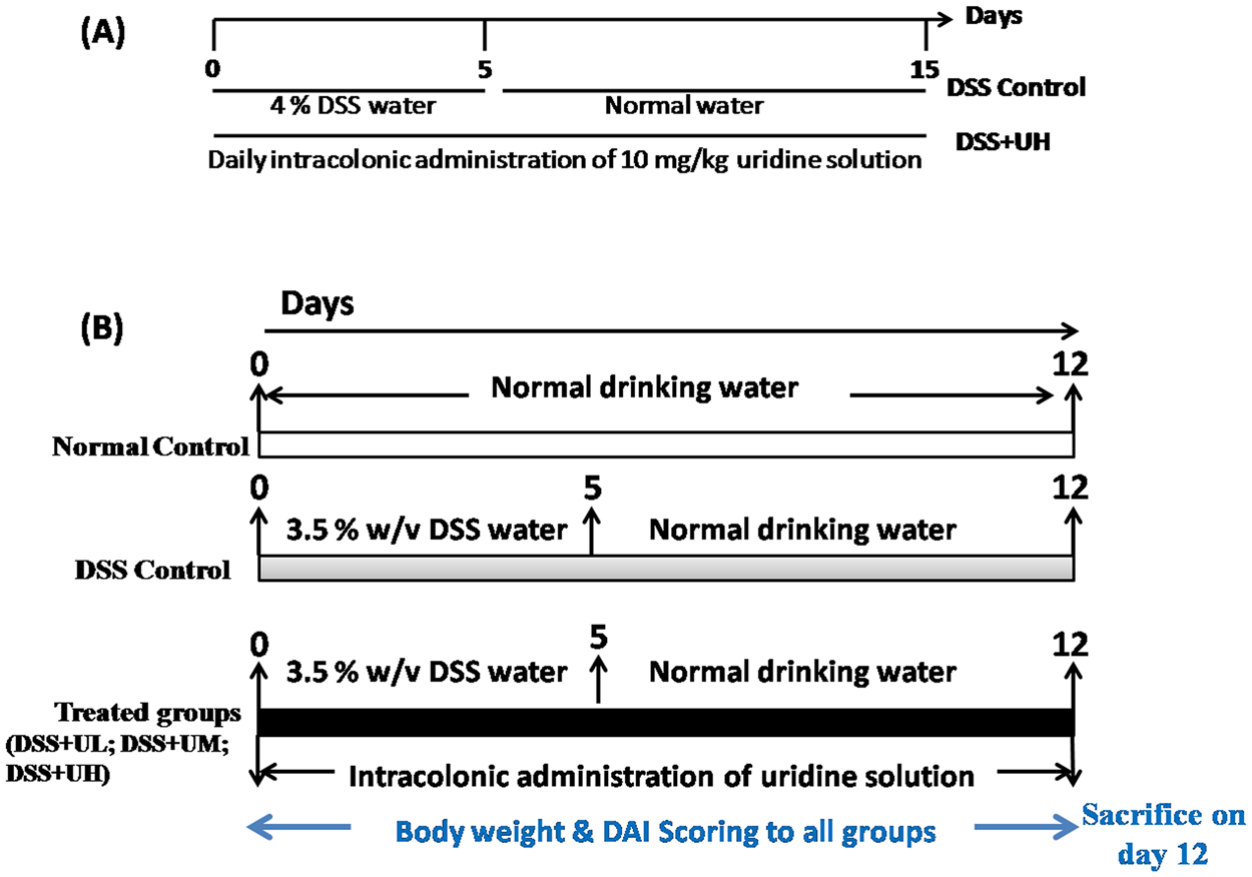
Schematic diagram to illustrate the experimental design. (**A**) Survival study: Mice were divided into two groups DSS control & DSS+ 10 mg/kg uridine treated group and challenged with 4% w/v of DSS with drinking water for 5 days, and were further assessed for survival upto day15. (**B**) Evaluation of dose dependent anti-inflammatory effect of intracolonic administered uridine on Dextran Sulfate Sodium (DSS)-induced experimental colitis in Mice; Experimental Colitis was induced by administration of 3.5% of w/v DSS with drinking water for 5 days and later on normal drinking water for next 7 days. Uridine treatment group received intracolonic uridine solution once daily from day 0 to day 12 while remaining group mice received normal saline. Body weight, health status were measured daily. Colon length measurement, colon histopathology were performed after necropsy. DSS+UL, Low dose uridine 1 mg/kg; DSS+UM, medium dose uridine 5 mg/kg; V, DSS+UH, higher dose uridine 10 mg/kg.

**Figure 2:**
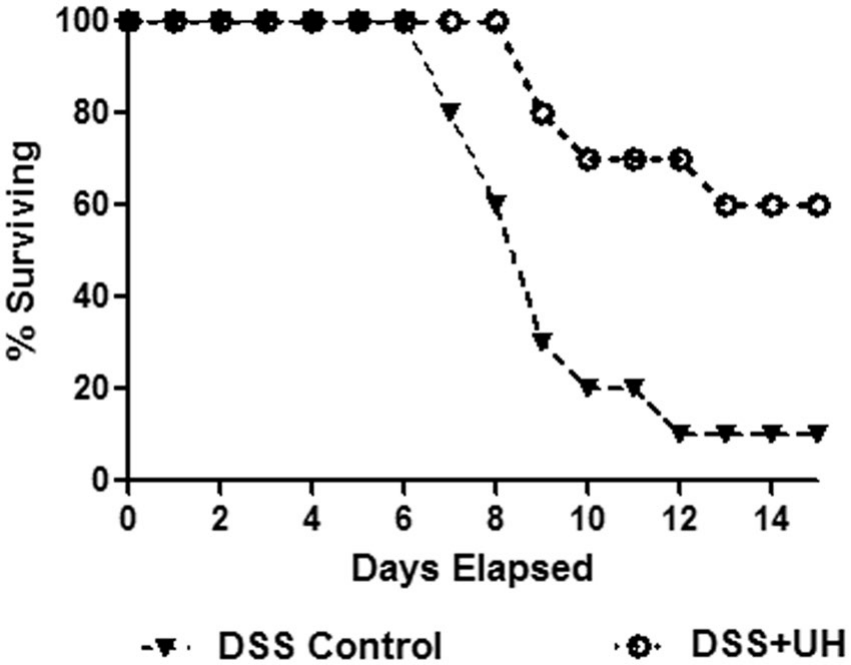
Survival study. Comparison of survival rate was done with mice challenged with 4% of DSS and DSS+ Uridine 10 mg/kg. Percentage survival data of n = 10 animals.

We used a higher concentration of DSS (4% w/v concentration) in order to study the effect of uridine on overall mortality of animals (Fig. 1A). A lower concentration of DSS (3.5% w/v) was used in all subsequent experiments to study the dose dependent anti-inflammatory effect of uridine (Fig. 1B). It was found that the survival rate was significantly prolonged in the uridine 10 mg/kg +DSS group (DSS+UH) compared to DSS control mice (Fig. 2). Only 10% of the DSS control mice survived while 60% of the DSS+UH treated mice survived up to 15 days.

### Body weight

**Figure 3:**
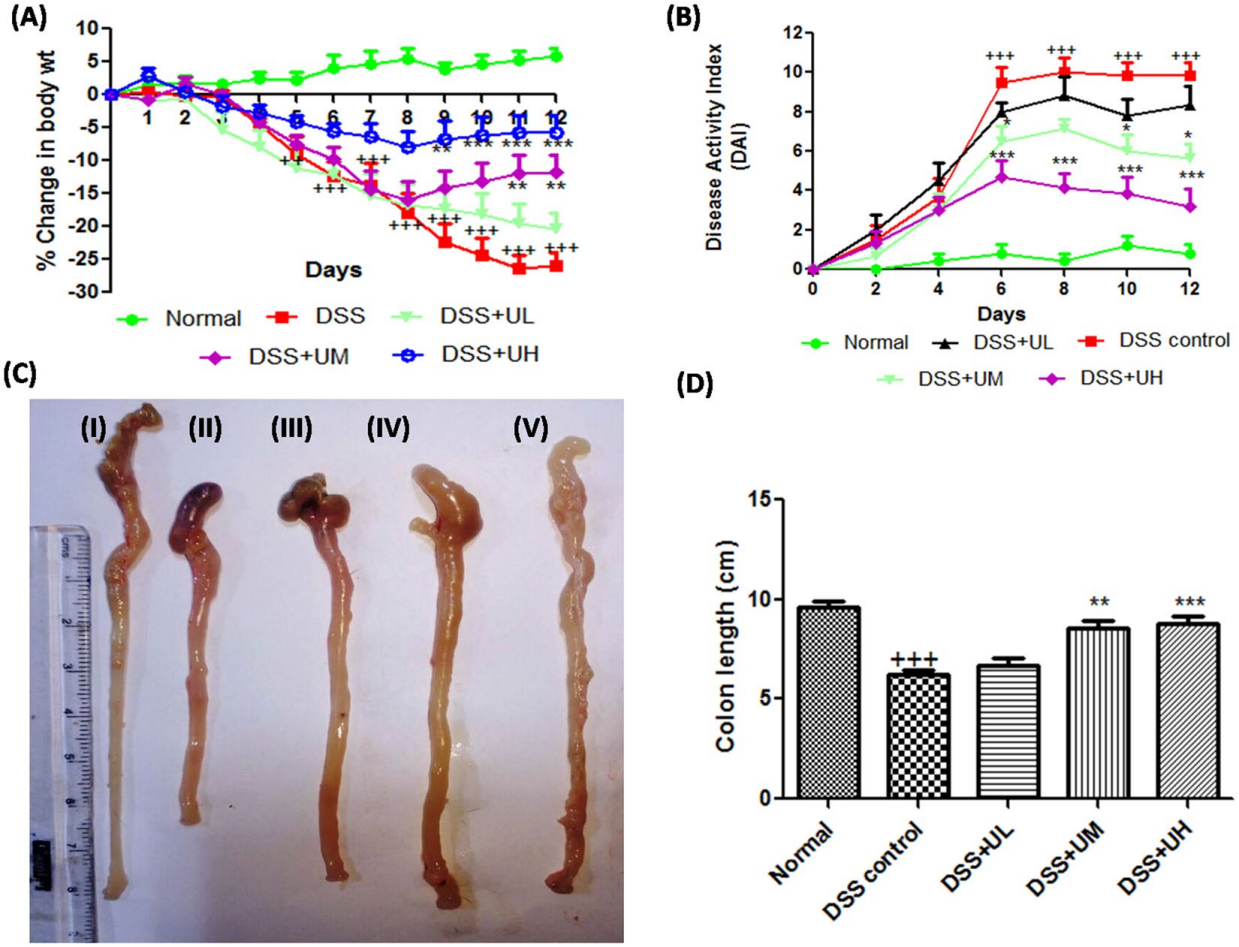
Effect of uridine treatment on the clinical signs of colitis and macroscopic signs of inflammation in colon tissue. Experimental colitis was induced by 3.5% w/v of DSS in drinking water (*ad libidum)* for 5 days. (**A**) Percentage change in body weight. (**B**) Disease activity index, a composite measure of weight loss, stool consistency and blood in stool. (**C**) Representative photographs showing colon tissue from I, Normal; II, DSS control; III, DSS+UL; IV, DSS+UM; V, DSS+UH (**D**) Changes in colon length. Data presented indicate the mean ± SEM (n = 6). ^+++^p < 0.001 vs Normal, *p < 0.05, **p < 0.01 and ***p < 0.001 vs DSS control.

All mice treated with 3.5% of DSS showed body weight loss starting from day 4 post DSS administration (Fig. 3A). Uridine treatment showed a protective effect on colitis-induced body weight loss. The uridine higher dose (10 mg/kg) group (DSS+UH) showed a significant improvement in body weight from day 9 to day 12 (p < 0.01 to p < 0.001) compared to DSS control group. The uridine medium dose (5 mg/kg) group (DSS+UM) showed a significant inhibition of weight loss from day 11 (p < 0.01) compared to the DSS control group. The uridine low dose (1 mg/kg; DSS+UL) treatment also showed slight improvement in % change in body weight when compared to DSS control group (p > 0.05).

### Disease Activity Index (DAI)

DAI, calculated as a composite of body weight loss, stool consistency and stool blood, was scored on alternate days to analyze the anti-inflammatory potential of uridine. DSS (3.5%) administration was associated with significant clinical changes including weight loss, appearance of occult faecal blood and diarrhoea in DSS control mice. The DAI of DSS control group was significantly elevated on day 4 compared to baseline and reached its maximum on day 8. Treatment with 5 and 10 mg/kg of uridine markedly reduced the DAI score from day 6 and onwards (Fig. 3B). Uridine treatment delayed or reduced the appearance of the colitis symptoms like appearance of hemoccult and diarrhoea which resulted in a significant reduction of DAI in DSS+UM and DSS+UH groups. The representative photos of DSS control mice showing rectal bleeding compared to DSS+UH mice and comparison of stool samples from each group on day 12 are shown in Supplementary Figs S1 and S2.

### Colon length

All colon tissues were collected and measured at the end of study, to study the effect of uridine on inflammation induced decrease in colon length, a classical symptom of colonic inflammation. Severe signs of inflammation and bleeding clearly observed in colon tissue of DSS control (Fig. 3C) were eliminated in DSS+UM and DSS+UH groups. The colon tissues of mice exposed to DSS exhibited a marked decrease in colon length compared with the normal group (Fig. 3D, p < 0.001). The colon of mice treated with DSS+UM and DSS+UH showed a significant (p < 0.01 & p < 0.01) improvement in colon length when compared to DSS control and almost completely reverted back to normal length. The lower dose of uridine (1 mg/kg) had no marked effect on colon length compared to DSS control.

### Histopathology

**Figure 4:**
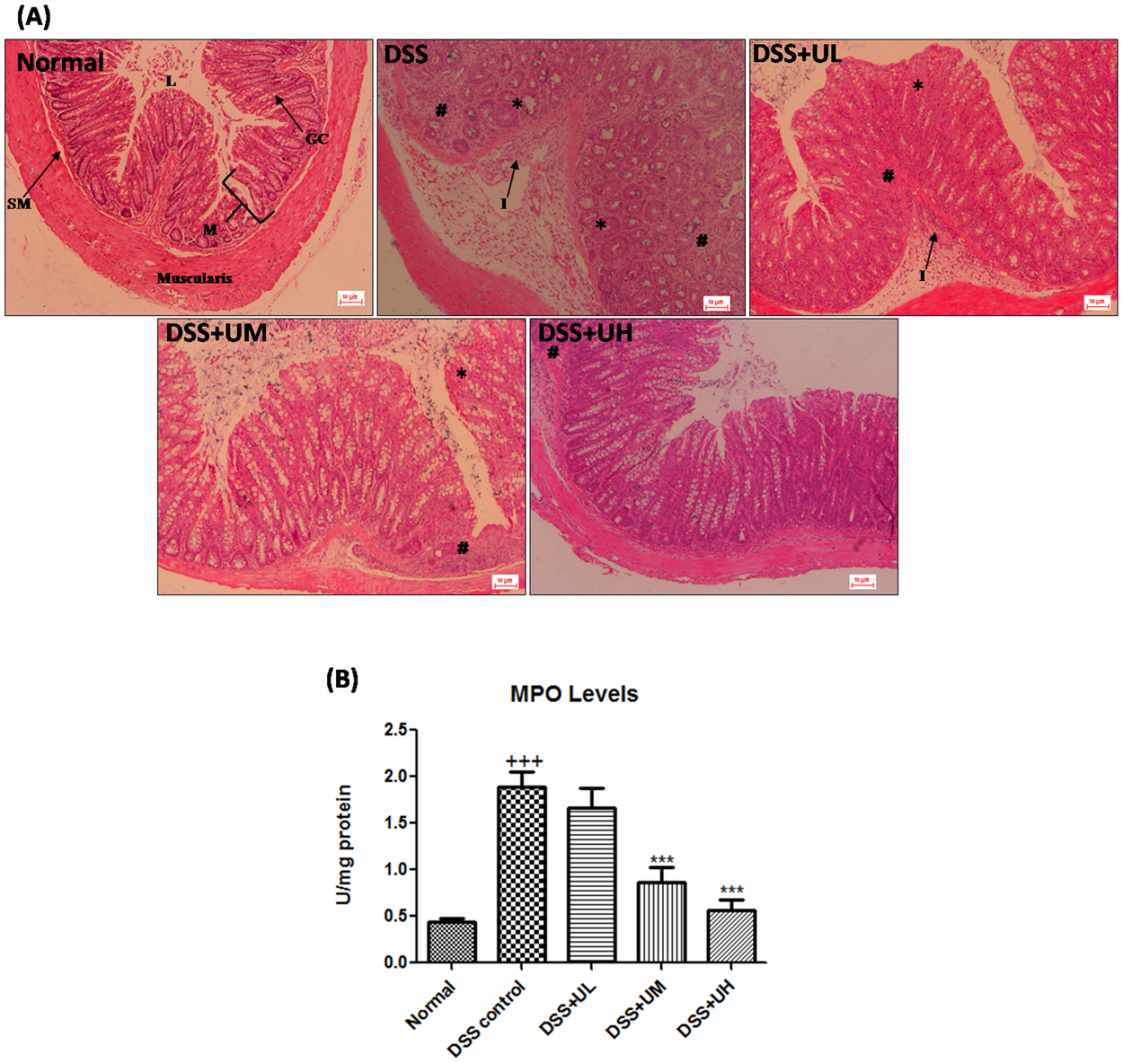
Effect of uridine treatment on the histopathological changes and myloperoxidase (MPO) activity of the colon tissue. (**A**) Representative images of hematoxylin and eosin staining of colon tissue from each group taken at 100x magnification. Colon tissue from Normal group did not show any pathological modification, DSS-induced colon tissue injury was associated with partial destruction of the epithelial architecture such as loss of crypts and epithelial integrity, submucosal edema and intense infiltration of inflammatory cells. Treatment with different dosages attenuated the injury of colon tissue in dose dependent manner. L, Lumen; GC, Goblet cells; M, Mucosa; SM, Submucosa; I, Inflammation; asterisk (*) indicates area of goblet cell depletion and distortion of crypt architecture; number sign (#) indicates cellular infiltration. (**B**) The myloperoxidase (MPO) activity in colon tissue, values are the mean ± SEM (n = 6). ^+++^p < 0.001 vs Normal and ***p < 0.001 vs DSS control.

Histological analysis (H&E staining) of the distal colon tissue revealed that treatment of mice with DSS leads to destruction of crypt structure with goblet cell loss, a disturbed epithelial layer and massive infiltration of inflammatory cells in the colon tissue when compared to the normal morphology of colon tissue (Fig. 4A). Colon tissue from DSS+UH treated mice exhibited predominantly intact colon histology, with reduced signs of inflammation, preserved epithelial layer and crypt structure when compared to DSS control group. The DSS+UL and DSS+UM treated mice showed moderate to mild signs of inflammation in colon histopathology.

### Myeloperoxidase (MPO) activity assay

Figure 4B depicts the MPO activity in colon tissue of mice of different groups. There was a significant (p < 0.001) increase in the MPO activity of colon tissue of DSS control group when compared to normal group. Uridine at 5 and 10 mg/kg significantly (p < 0.001) attenuated DSS-induced increase in levels of MPO when compared to DSS control group. 1 mg/kg uridine was unable to exert a significant effect on MPO activity.

### Serum cytokine levels

**Figure 5:**
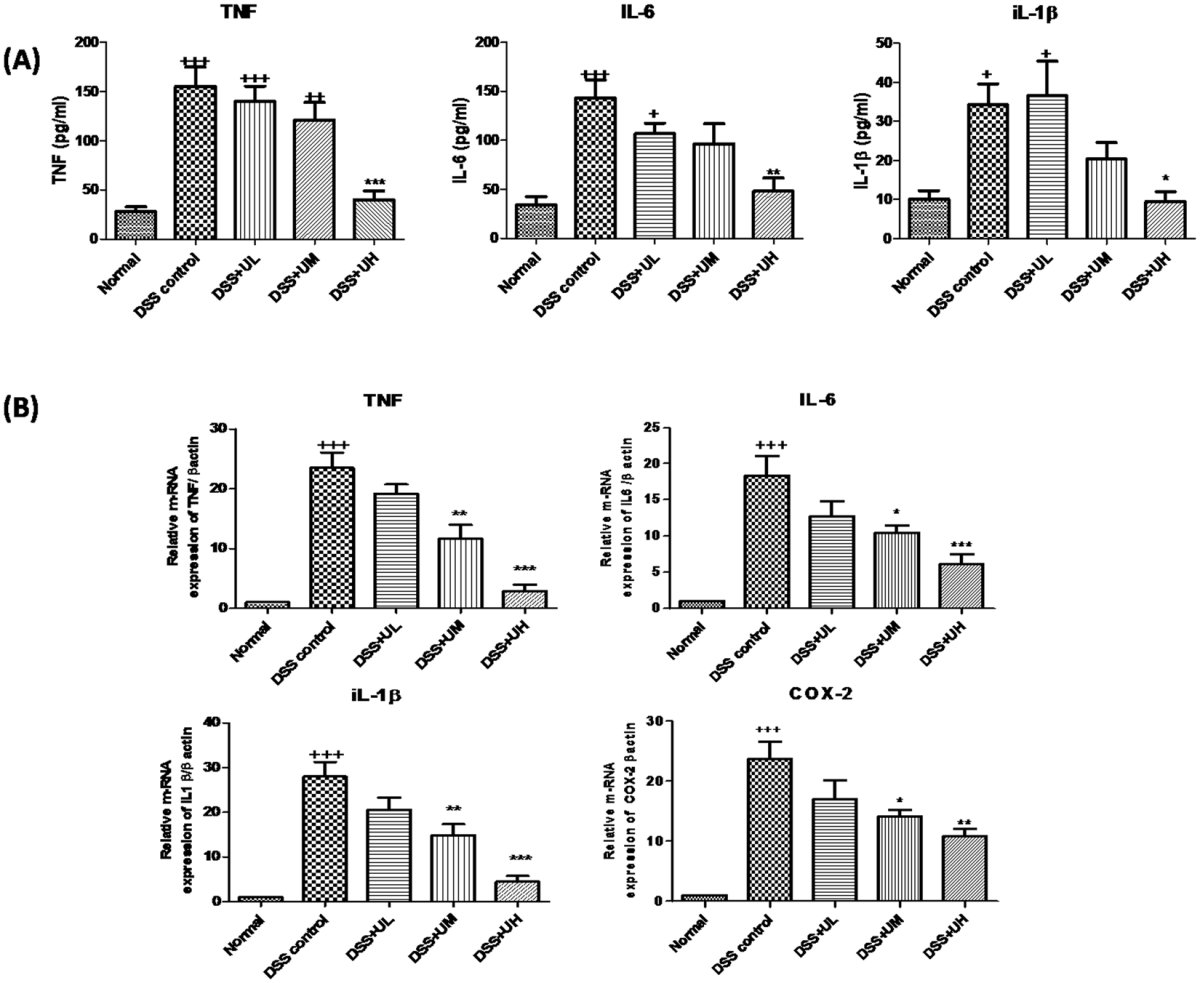
Effect of uridine treatment on the inflammatory biomarkers of DSS induced UC. (**A**) Serum proinflammatory cytokines level TNF, IL-6 and IL-1β measured by cytometric bead array assay. (**B**) m-RNA expression of pro-inflammatory cytokines and enzyme in colon tissue measured by RT-PCR. All the values were expressed as mean mean ± SEM (n = 5). ^+++^p < 0.001 vs Normal, *p < 0.05, **p < 0.01 and ***p < 0.001 vs DSS control.

Serum levels of pro-inflammatory cytokines TNF-α, IL-6 and IL-1β were estimated by flow cytometry and significantly elevated levels of these cytokines were found in DSS control group (p < 0.001) compared to normal group (Fig. 5A). Uridine high dose treatment group (DSS+UH) showed a significant decrease in the levels of TNF (p < 0.001), IL-6 (p < 0.01) and IL-1β (p < 0.05) compared to DSS control. A slight decrease in the levels of these cytokines was also observed in DSS+UM group. The lower dose of uridine had no effect on the levels of cytokines.

### Cytokine/chemokine mRNA expression in the colon

As shown in Fig. 5B, a significant (p < 0.001) increase in the mRNA expression of pro-inflammatory cytokines, TNF-α, IL-6 and IL-1β was observed in the DSS control mice when compared with normal group mice. Treatment with uridine at 5 and 10 mg/kg doses significantly reversed the changes in cytokine levels when compared to the DSS control mice (TNF and IL-1β: p < 0.01 at 5 and p < 0.001 at 10 mg/kg uridine; IL-6: p < 0.05 at 5 mg/kg and p < 0.001 at 10 mg/kg uridine). Similarly, the mRNA levels of COX-2, another pro-inflammatory mediator, were significantly (p < 0.001) increased in colon tissues of DSS control mice when compared to normal group mice. Treatment with 5 and 10 mg/kg of uridine significantly decreased the COX-2 expression when compared to the DSS control mice. (p < 0.05 at 5 mg/kg and p < 0.01 at 10 mg/kg uridine).

### Estimation of protein expression in the colon

**Figure 6:**
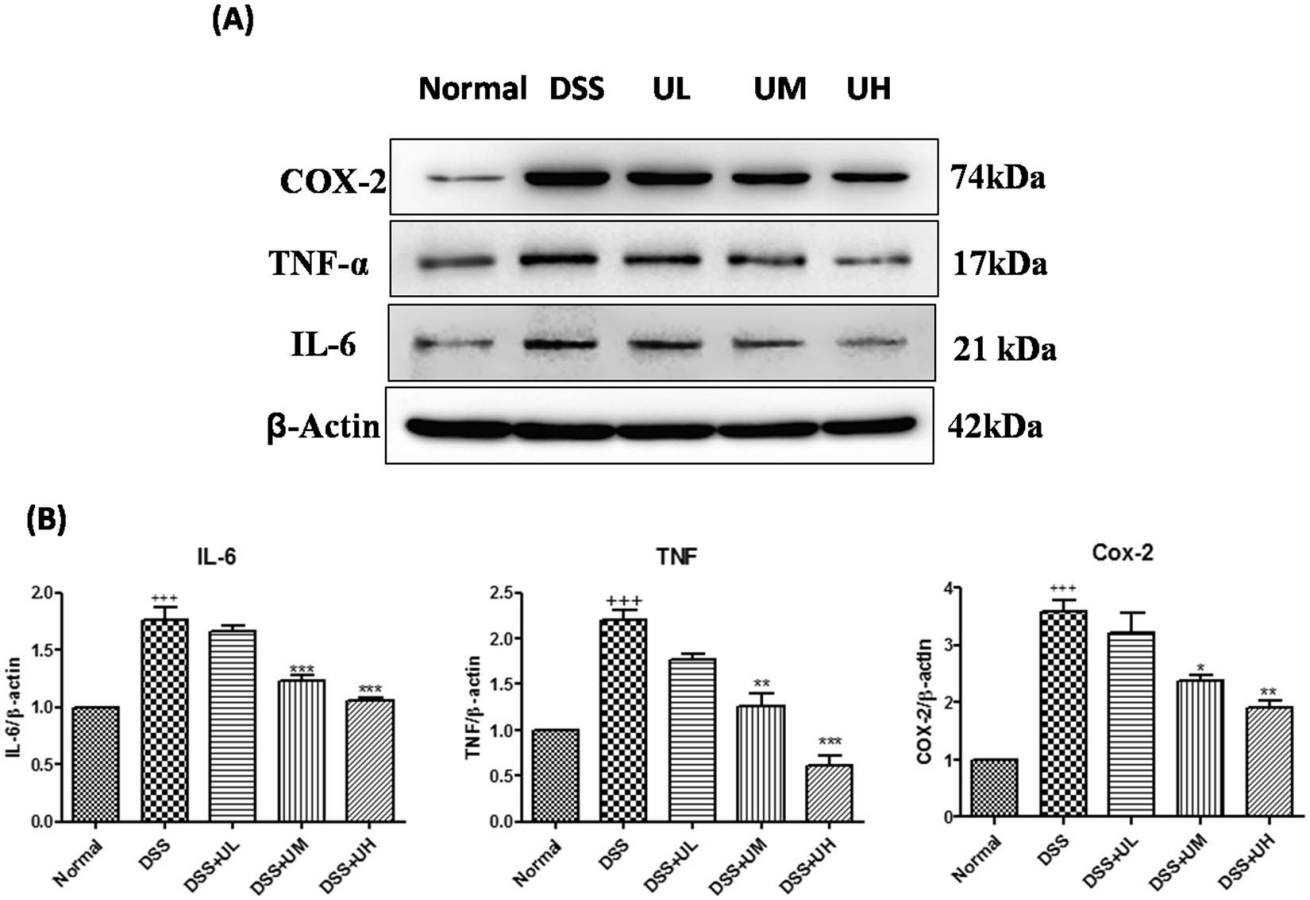
Effect of uridine treatment on the protein expression of pro-inflammatory cytokines, enzyme levels in colon tissue. (**A**) Representative western blots showing changes in the protein expression of pro-inflammatory cytokines and enzyme, TNF, IL-6, and COX-2 in colon tissue (**B**) Graphical depiction of western blotting analysis showing quantitative results. Band intensities were quantified using NIH Image J software. The relative protein levels were normalized to the b-actin level. Each value was calculated on the basis of the data obtained from three independent experiments. Values are the mean ± SEM (n = 3). ^+++^p < 0.001 vs Normal, *p < 0.05, **p < 0.01 and ***p < 0.001 vs DSS control.

Protein expression of the proinflammatory cytokines TNF, IL-6, and COX-2 enzyme was estimated in the colon tissue using immunoblotting (Fig. 6A). There was a significantly (p < 0.001) increased expression of these proteins in colon tissue of DSS control mice compared to normal mice (Fig. 6B). The elevated levels of TNF, IL-6, were significantly reduced in the colon tissue of DSS+UM and DSS+UH groups when compared to DSS control group (p < 0.01; p < 0.001).

**Figure 7:**
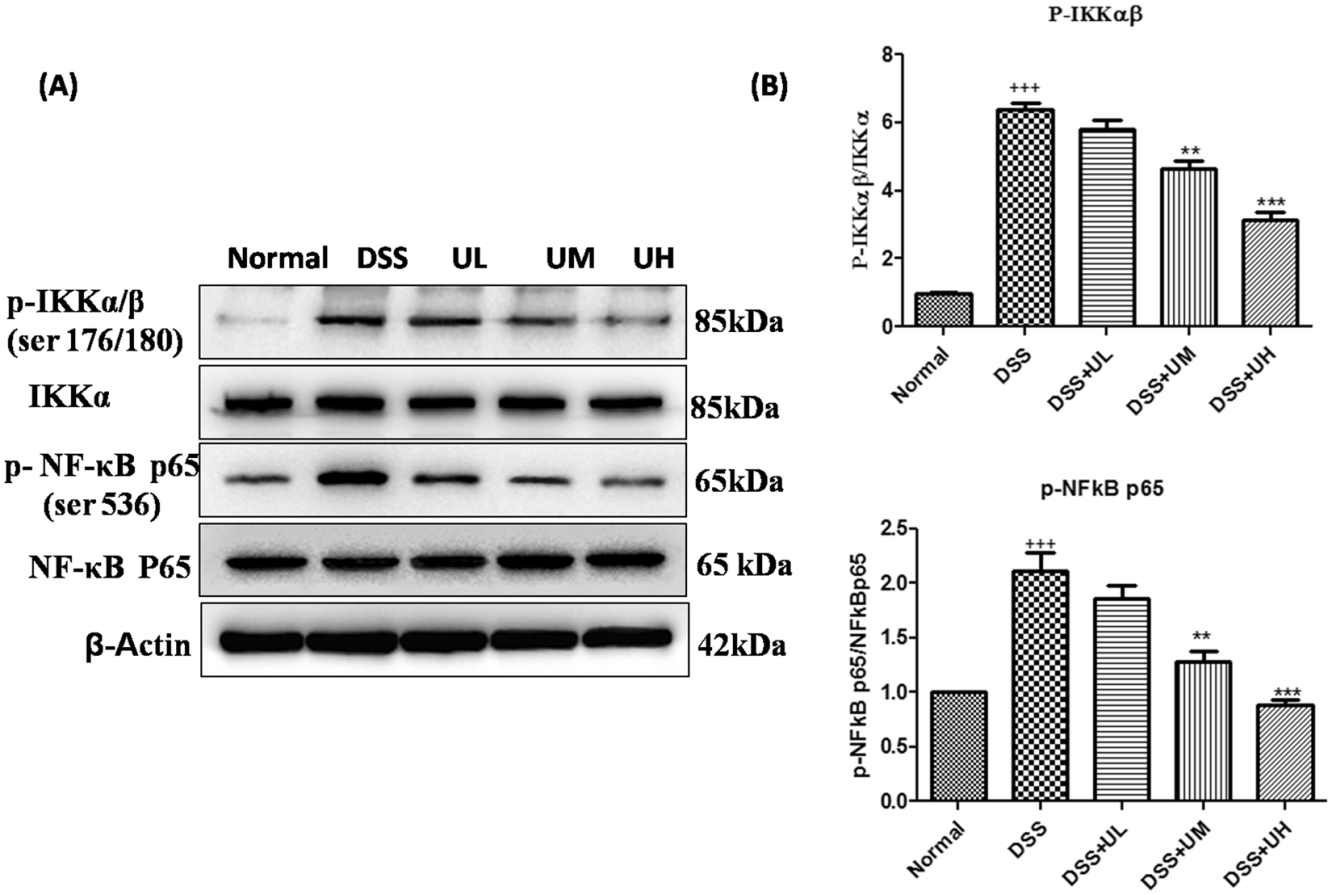
Uridine treatment inhibits DSS induced NF-κB activity in colon tissue. (**A**) Representative western blotting bands showing the expression levels of phospho-NF-κB-p65, NFκB and phospho-IKKα/β (176/180), IKKα/β (176/180). β-actin was used for equal loading of protein. (**B**) Bar diagrams showing the relative ratio of each protein. All the values were expressed as mean mean ± SEM (n = 3). ^++^p < 0.01, ^+^p < 0.05 vs Normal, *p < 0.05, **p < 0.01 and ***p < 0.001 vs DSS control.

Treatment with 5 mg/kg and 10 mg/kg of uridine significantly reduced the levels of COX-2 compared to DSS control (P < 0.05 at 5 mg/kg and p < 0.01 at 10 mg/kg of uridine). The lower dose of uridine (1 mg/kg) was unable to exert any significant effect on these protein levels compared to DSS control. Further, the phosphorylation of IKKα/β and NF-κB (ser536) was investigated to evaluate DSS augmented NF-кB transcriptional activation in the colon tissue (Fig. 7A). It was observed that phosphorylation of IKKα/β and NF-κB was significantly (p < 0.001) increased in DSS control group compared to normal mice (Fig. 7B). There was a dose-dependent decrease in the phosphorylation of NF-κB in the uridine treated groups with respect to DSS control group. Phosphorylation of IKKα/β was significantly (p < 0.01; p < 0.001) reduced with 5 and 10 mg/kg of uridine compared to DSS control and almost reverted back to the normal levels. However, uridine treatment at 1 mg/kg dose was unable to exert any significant effect.

## Discussion

Uridine has attracted attention as a novel therapeutic modulator of inflammation due to proven efficacy in various animal models of inflammation combined with a good safety profile^21, 24^. However, poor oral bioavailability has limited the use of uridine as an effective oral therapeutic agent^25^. We have previously reported that locally administered uridine has potent anti-inflammatory effects in animal models of lung inflammation and arthritis^21, 22^. The present study is the first of its kind demonstrating the efficacy of intracolonic administration of uridine in experimental DSS induced ulcerative colitis. Administration of 3.5% DSS to mice for 5 days elicited the predominant clinical symptoms of colitis including weight loss, diarrhoea, bloody faeces, crypt distortion, epithelial injury, reduced colon length and inflammatory cell infiltration. These clinical symptoms of IBD were efficiently relieved by the treatment of uridine in a dose dependent manner. The higher and medium dose of uridine significantly reduced the DAI score, macroscopically visible damage and reversed inflammation induced reduction in colon length. In the survival study the 10 mg/kg uridine treatment prolonged the survival rate of mice compared to DSS control group. These observations clearly suggested that uridine at 10 mg/kg is efficient in suppressing overt clinical features of DSS induced colitis.

Uridine treatment resulted in a dose-dependent protection of histological integrity in the colon tissue. Since DSS-induced mice developed immunological abnormalities, such as prominent shortening of the colon, thickening of the muscular layer and crypt damage in the inflamed areas and inflammatory cell infiltration in the lamina propria and mucosa of the colon and that was noticeably reduced by the uridine treatment. In this study increased MPO levels, an accepted measure of neutrophil infiltration, were substantially reduced by uridine treatment demonstrating a significant reduction in the infiltration of neutrophils. These results are in line with our previous studies where we have shown that uridine inhibits leukocyte adhesion *in vitro* ^26^ and local administration of uridine reduced neutrophil influx in BAL fluid in animal model of lung inflammation^21^ and almost abrogated neutrophil and macrophage influx in synovium of antigen induced arthritic mice^22^.

The pro-inflammatory cytokines such as TNF, IL-6 and IL-1β have a pivotal role in the pathogenesis of IBD^9, 27^. Several studies have also reported that interaction between pro-inflammatory cytokines and the intestinal mucosal immune system can lead to the disruption of tight junction proteins and affect intestinal homeostasis^28^. Increased serum and tissue levels of pro-inflammatory cytokines such as IL-6, IL-1β and TNF-α are characteristic features of colitis and many other chronic inflammatory diseases^29^. Secretion of IL-6, IL-1β and TNF was significantly elevated in the serum by DSS treatment and was remarkably suppressed by the uridine treatment at a higher dose. This is noteworthy because a single intraarticular injection of uridine that protected mice from antigen-induced arthritis did not inhibit the rise of proinflammatory cytokines in serum^22^.

We have previously shown that uridine inhibited the extravasation of inflammatory cells into the synovium and inhibited expression of TNF, IL-6 and adhesion molecules in the synovium of arthritic mice^22^. Uridine treatment inhibited the increased mRNA expression for TNF, IL-1, IL-6 and protein levels of TNF and IL-6 in a dose dependent manner. Taken together, our results suggest that uridine was able to decrease the levels of these pro-inflammatory cytokines and the inhibitory effect of uridine is already at the mRNA level affecting both local and circulating cytokine levels.

Upregulation of inducible pro-inflammatory enzyme COX-2 in the intestinal epithelium of IBD patients is well documented and it plays an important role in the amplification of mucosal inflammation in IBD^30, 31^. Our results indicated that increased expression of COX-2 was attenuated at both mRNA and protein level by uridine treatment. It could be speculated that uridine might also exert an analgesic effect due to the observed effect on COX-2 expression. However, this needs to be studied in detail in specific models of pain and analgesia.

The synthesis of COX-2 as well as production of proinflammatory cytokines including TNF and IL-6 is directly modulated by the pleiotropic transcription factor NF-κB^32, 33^. Phosphorylation of IKKα/β (ser 176/180) is a critical step in NF-κB activation and the expression of P-IKKα/β (ser 176/180) was found to be markedly increased in DSS control group^31^. Our results clearly indicated that treatment of uridine at medium and high dose was able to inhibit phosphorylation of IKKα/β without affecting the expression of IKKα. Further, reduced levels of P-NF-κB p65 in colon tissue of uridine treated groups (DSS+UM & DSS+UH) confirmed that uridine attenuates NF-kB activation. Thus, our data strongly suggests that the anti-ulcerative colitis effect of uridine may be associated with the suppression of NF-κB signaling activation.

In summary, the findings in our study suggest that intra-colonic administration of uridine effectively prevented the development and progression of the DSS induced colitis symptoms in C57 BL6 mice. We highlighted that the plausible underlying mechanism of uridine might associated with alleviating inflammatory responses, inhibiting NF-κB signaling activation and reducing the neutrophils infiltration. Collectively, our results suggest that uridine has the potential to serve an as effective anti-IBD therapy. Further it would be of great interest to investigate therapeutic benefit of uridine treatment in already established IBD.

### Materials

Dextran sulphate sodium (DSS; product code# 02160110; Mw: 36000–50000) and uridine (product code# 02103216) was purchased from MP Biomedicals, Santa Ana, CA, USA.

### Animals

Male C57BL/6 mice, 7–8 weeks of age were purchased from Sanzyme P. Ltd. Banjara Hills, Hyderabad, India. All mice were housed and fed in a dedicated pathogen-free facility and maintained at standard laboratory conditions of 23 ± 2 °C under 12-hour day and night cycles throughout the experiment. All procedures described were reviewed and approved by the Institutional Animal Ethics Committee (IAEC), NIPER Hyderabad, India. The animal experiments were conducted in accordance with the CPCSEA guidelines (IAEC approval number: NIP/12/2015/PC/162).

### Induction of colitis and treatment protocol

The DSS model is a robust, well established model and has been used to screen potential drug candidates and to investigate their mechanism of action^26^. Mice were randomly divided into five groups (n = 6), comprising a normal group (Normal), DSS control, DSS and low dose uridine (1 mg/kg body weight; DSS+DSS+UL), DSS and medium dose uridine (5 mg/kg; DSS+UM), DSS and high dose uridine (10 mg/kg; DSS+UH;). Experimental colitis was induced in mice by administration of 3.5% w/v DSS in drinking water for 5 days, followed by a regime of 7 days of fresh water (reflecting acute inflammation), whereas the normal group received only normal drinking water throughout the experiment^34^. In present study, uridine was administered intra-colonially, thus avoiding bioavailability problems associated with oral or intravenous administration. Daily instillation of 0.1 ml intracolonic saline in the control group or appropriate dose of uridine in the treatment group was performed on anesthetized mice by using flexible polyethylene tubing positioned in the midcolon after lubrication (4 cm proximal to the anus). Uridine treatment group received intracolonic uridine solution once daily for 12 days starting on day 0 of the DSS treatment. At day 12, mice were euthanized under CO_2_ asphyxiation and the colon was removed; length was measured and stored for subsequent analysis.

For the survival study, 10 mice each in the DSS and DSS+UH groups were subjected to 4% w/v of DSS until day 5, when they were returned to normal distilled water and monitored for survival upto day 15^35^.

### Evaluation of Colitis

Body weight change, stool consistency and gross bleeding were assessed daily. Collection of feces was done by placing a single mouse in an empty cage without bedding material for 15–30 min and fecal pellet was used to monitor hemoccult. The criteria to calculate disease activity index (DAI) score described previously^36^ are shown in Table 1.

**Table 1:** The criteria for DAI scoring.

Weight Loss		Stool consistency		Hemoccult	
Range	Score	Criteria	Score	Criteria	Score
None	0	**Well form pellets**	0	**Negative**	0
1–5%	1				
5–10%	2	**Loose stool**	2	**Positive**	2
10–20%	3				
>20%	4	**Diarrhoea**	4	**Gross bleeding**	4

The disease activity index (DAI) value is calculated ashe DAI value is calculated as the sum of the scores for weight loss, stool consistency, and blood in feces.

### Histology

Colon tissue samples were fixed in 10% neutral buffered formalin and embedded in paraffin. 5 µm thick sections were prepared from each block, stained with haematoxylin & eosin and observed under phase contrast microscope (100x magnification, Nikon, Japan).

### Myeloperoxidase (MPO) activity assay

MPO activity as a measure of neutrophil infiltration is a well established biomaffigrker of ulcerative colitis^37, 38^. Tissue MPO activity was performed as reported previously with some modifications^39^. Briefly, colon tissue was thawed and homogenized in 50 mM phosphate buffer (pH 6) containing 0.5% hexadecyltrimethylammonium bromide (HTAB). The tissue homogenates were subjected to a brief sonication for 10 s, one cycle of freezing, thawing and sonicated for a further 10 s. After sonication, suspensions were centrifuged at 13,000 rpm for 20 min at 4 °C. The supernatant (0.1 mL) was mixed in 2.9 ml of in 50 mM phosphate buffer (pH 6) containing 0.53 mM of o-dianisidine hydrochloride and 0.15 mM hydrogen peroxide and the change in absorbance was measured every 15 s for, 5 min at 460 nm. The results were expressed in units (U) of MPO/ mg of protein. The protein concentrations of each sample were evaluated by using the Bradford methodusing bovine serum albumin as the standard.

### Flow Cytometry

Serum samples were processed for cytokine measurement using Cytometric Bead Array (CBA Flex Sets BD Biosciences, UK). Three key cytokines were assessed: IL-1β, IL-6 and TNF. The detection limit for IL-1β, IL-6 and TNF was 1.9 pg/ml, 1.4 pg/ml and 2.8 pg/ml respectively.

### RT-PCR analysis

For quantitative real time PCR measurements, colon tissue samples were cut and frozen immediately in liquid nitrogen with TRI reagent (Sigma Aldrich) and stored at −80 °C until RNA extraction. Total RNA isolation was performed as reported earlier^40^. Sample quality control and the quantitative analysis were carried out by NanoDrop (Thermo Scientific). Reverse-transcription to cDNA was performed using Verso cDNA synthesis kit according to the manufacturer’s instructions. The Real-Time PCR reaction was performed on the ABS 7500 fast instrument with SYBR Green PCR master mix (Applied Biosystems, Foster City, CA, USA) with an initial denaturation step at 95 °C for 10 min, followed by 40 cycles of 95 °C for 10 s annealing temperature with extension step for 45 s at 55 °C by using respective following primers of various target genes: TNF-Left primer (L): 5′-GAACTGGCAGAAGAGGCACT-3′, TNF-Right primer (R): 5′-AGGGTCTGGGCCATAGAACT-3′, IL-1β (L): 5′-GCCCATCCTCTGTGACTCAT-3′, IL-1β(R): 5′-AGGCCACAGGTATTTTGTCG-3′, IL-6(L): 5′-AGTTGCCTTCTTGGGACTGA-3′, IL-6 (R): 5′-CAGAATTGCCATTGCACAAC-3′, β-Actin(L): 5′-AGCCATGTACGTAGCCATCC-3′ and β-Actin(R): 5′-CTCTCAGCTGTGGTGGTGAA-3′. The relative changes in TNF-α, IL-1 β, and IL-6 and COX-2 with respect to β-Actin expression were examined using 2^−ΔΔCt^ method using normal mice as reference.

### Western blot analysis

Colon tissues were cut and frozen immediately in liquid nitrogen and stored at −80 °C. Protein lyaste were prepared as described previously^41^. Briefly, Colon tissue was homogenized in RIPA buffer with 1% protease and phoshphatase inhibitor cocktail (Sigma-Aldrich, St. Louis, MO, USA) and incubated on ice for 1 h. Lysates were then centrifuged for 15 min at 13000 rpm at 4 °C. Proteins were separated using SDS–PAGE and transferred onto PVDF (poly-vinylidene difluoride) membrane. The blots were blocked with 3.5% BSA (Bovine Serum Albumin) in TBST (20 mM Tris–HCl, pH 7.4, 137 mM NaCl, and 0.05%Tween-20) at room temperature for 1 h and incubated overnight with the appropriate primary antibody at 4 °C. After washing with TBST, the blots were incubated with peroxidase conjugated secondary antibody for 1 h. β-actin was used as an internal control to ensure equal protein loading. Bands were monitored using enhanced chemiluminescence reagent (Millipore, U.S.A). The strength of western blotting bands was determined by Image J density measurement program.

Antibodies used for western blot analysis were PNF-κB (p65), NF-κB P65, phospho-Iκβα, IκBα, COX-2, TNF-α, IL-6, IL-10 and MPO. All the primary and secondary antibodies were obtained from Cell Signaling Technology, Baverly, USA.

### Statistical analysis

All data are represented as mean ± SEM. Statistical analysis was performed using GraphPad Prism 5.0 software, utilizing One-way ANOVA and Dunnett’s multiple comparison test and considered significant if p values were <0.05.

## Electronic supplementary material

supplementary figures

## References

[1] Baumgart DC, Carding SR (2007). Inflammatory bowel disease: cause and immunobiology. The Lancet.

[2] Bouma G, Strober W (2003). The immunological and genetic basis of inflammatory bowel disease. Nature Reviews Immunology.

[3] Baumgart DC, Sandborn WJ (2012). Crohn’s disease. The Lancet.

[4] Xavier R, Podolsky D (2007). Unravelling the pathogenesis of inflammatory bowel disease. Nature.

[5] Stenson, W. F., Tremaine, W. J. & Cohen, R. D. Ulcerative Colitis: Clinical Manifestations and Management. *Yamada’s Atlas of Gastroenterology*, 216–224 (2016).

[6] Kane SV, Cohen RD, Aikens JE, Hanauer SB (2001). Prevalence of nonadherence with maintenance mesalamine in quiescent ulcerative colitis. The American journal of gastroenterology.

[7] Brown SJ, Mayer L (2007). The immune response in inflammatory bowel disease. The American journal of gastroenterology.

[8] Sartor RB (2006). Mechanisms of disease: pathogenesis of Crohn’s disease and ulcerative colitis. Nature clinical practice Gastroenterology & hepatology.

[9] Nenci A (2007). Epithelial NEMO links innate immunity to chronic intestinal inflammation. Nature.

[10] Ulbrich H, Eriksson EE, Lindbom L (2003). Leukocyte and endothelial cell adhesion molecules as targets for therapeutic interventions in inflammatory disease. Trends in pharmacological sciences.

[11] Friswell M, Campbell B, Rhodes J (2010). The Role of Bacteria in the Pathogenesis of Inflammatory Bowel. Liver.

[12] Papadakis KA, Targan SR (2000). Role of cytokines in the pathogenesis of inflammatory bowel disease. Annual review of medicine.

[13] Reaves TA, Chin AC, Parkos CA (2005). Neutrophil transepithelial migration: role of toll-like receptors in mucosal inflammation. Memórias do Instituto Oswaldo Cruz.

[14] Ferenczi, S., Szegi, K., Winkler, Z., Barna, T. & Kovács, K. J. Oligomannan Prebiotic Attenuates Immunological, Clinical and Behavioral Symptoms in Mouse Model of Inflammatory Bowel Disease. *Scientific Reports* **6** (2016). 10.1038/srep34132 PMC5034233 27658624

[15] Melgar S (2008). Validation of murine dextran sulfate sodium-induced colitis using four therapeutic agents for human inflammatory bowel disease. International immunopharmacology.

[16] Perše, M. & Cerar, A. Dextran sodium sulphate colitis mouse model: traps and tricks. *BioMed Research International* **2012** (2012). 10.1155/2012/718617 PMC3361365 22665990

[17] Nell S, Suerbaum S, Josenhans C (2010). The impact of the microbiota on the pathogenesis of IBD: lessons from mouse infection models. Nature Reviews Microbiology.

[18] Wang X (2016). Oroxyloside prevents dextran sulfate sodium-induced experimental colitis in mice by inhibiting NF-κB pathway through PPARγ activation. Biochemical pharmacology.

[19] Oh JY (2007). Protective effect of uridine on cornea in a rabbit dry eye model. Investigative ophthalmology & visual science.

[20] Müller T (2010). Local administration of uridine suppresses the cardinal features of asthmatic airway inflammation. Clinical & Experimental Allergy.

[21] Evaldsson C, Ryden I, Uppugunduri S (2007). Anti-inflammatory effects of exogenous uridine in an animal model of lung inflammation. International immunopharmacology.

[22] Narendra SC, Chalise JP, Magnusson M, Uppugunduri S (2015). Local but Not Systemic Administration of Uridine Prevents Development of Antigen-Induced Arthritis. PloS one.

[23] Uppugunduri S, Gautam C (2004). Effects of uridine, isomatitol and 4-thiouridine on *in vitro* cell adhesion and *in vivo* effects of 4-thiouridine in a lung inflammation model. International immunopharmacology.

[24] Van Groeningen G, Peters G, Nadal J, Laurensse E, Pinedo H (1991). Clinical and Pharmacologic Study of Orally Administrated Uridine. Journal of the National Cancer Institute.

[25] Pizzorno G (2002). Homeostatic control of uridine and the role of uridine phosphorylase: a biological and clinical update. Biochimica et Biophysica Acta (BBA)-Molecular Basis of Disease.

[26] Chassaing, B., Aitken, J. D., Malleshappa, M. & Vijay‐Kumar, M. Dextran sulfate sodium (DSS)‐induced colitis in mice. *Current Protocols in Immunology*, 15.25.11–15.25.14 (2014). 10.1002/0471142735.im1525s104 PMC3980572 24510619

[27] Schwanke RC (2013). Oral administration of the flavonoid myricitrin prevents dextran sulfate sodium‐induced experimental colitis in mice through modulation of PI3K/Akt signaling pathway. Molecular nutrition & food research.

[28] Abraham C, Medzhitov R (2011). Interactions between the host innate immune system and microbes in inflammatory bowel disease. Gastroenterology.

[29] Műzes G, Molnár B, Tulassay Z, Sipos F (2012). Changes of the cytokine profile in inflammatory bowel diseases. World J Gastroenterol.

[30] Singer II (1998). Cyclooxygenase 2 is induced in colonic epithelial cells in inflammatory bowel disease. Gastroenterology.

[31] Jurjus A (2016). Inflammatory bowel disease, colorectal cancer and type 2 diabetes mellitus: The links. BBA clinical.

[32] Chen C-C, Sun Y-T, Chen J-J, Chiu K-T (2000). TNF-α-induced cyclooxygenase-2 expression in human lung epithelial cells: involvement of the phospholipase C-γ2, protein kinase C-α, tyrosine kinase, NF-κB-inducing kinase, and I-κB kinase 1/2 pathway. The Journal of Immunology.

[33] Thummuri D (2015). Thymoquinone prevents RANKL-induced osteoclastogenesis activation and osteolysis in an *in vivo* model of inflammation by suppressing NF-KB and MAPK Signalling. Pharmacological Research.

[34] Liu T (2015). Treatment of dextran sodium sulfate-induced experimental colitis by adoptive transfer of peritoneal cells. Scientific reports.

[35] Li B, Alli R, Vogel P, Geiger TL (2014). IL-10 modulates DSS-induced colitis through a macrophage–ROS–NO axis. Mucosal immunology.

[36] Sahu BD, Kumar JM, Sistla R (2016). Fisetin, a dietary flavonoid, ameliorates experimental colitis in mice: Relevance of NF-κB signaling. The Journal of nutritional biochemistry.

[37] Muthas D (2016). Neutrophils in ulcerative colitis: A review of selected biomarkers and their potential therapeutic implications. Scandinavian Journal of Gastroenterology.

[38] Masoodi, I. *et al*. Biomarkers in the management of ulcerative colitis: a brief review. *GMS German Medical Science* **9** (2011). 10.3205/000126 PMC3046642 21394194

[39] Jeengar MK (2014). Improvement of bioavailability and anti-inflammatory potential of curcumin in combination with emu oil. Inflammation.

[40] Jeengar, M. K., Shrivastava, S., Veeravalli, S. C. M., Naidu, V. & Sistla, R. Amelioration of FCA induced arthritis on topical application of curcumin in combination with emu oil. *Nutrition* (2016). 10.1016/j.nut.2016.02.009 27178879

[41] Jeengar MK (2016). Emu oil based nano-emulgel for topical delivery of curcumin. International journal of pharmaceutics.

