# Supplementary material for: Uridine Ameliorates Dextran Sulfate Sodium (DSS)-Induced Colitis in Mice
Source: Sci Rep. 2017 Jun 20;7:3924. doi: 10.1038/s41598-017-04041-9 (PMC5478663; doi:10.1038/s41598-017-04041-9)

**Uridine Ameliorates Dextran Sulfate Sodium (DSS)-Induced Colitis in Mice**

**Manish Kumar Jeengar[1,2,*], Dinesh Thummuri[2], Mattias Magnusson[1], V.G.M. Naidu[2,3,$], Srinivas Uppugunduri [4,$]**

# SupplementaryFigures

**Supplementary figure S1.** Representative photo showing rectal bleeding in DSS control mice as compared to  mice from DSS+UH group on day 12

.

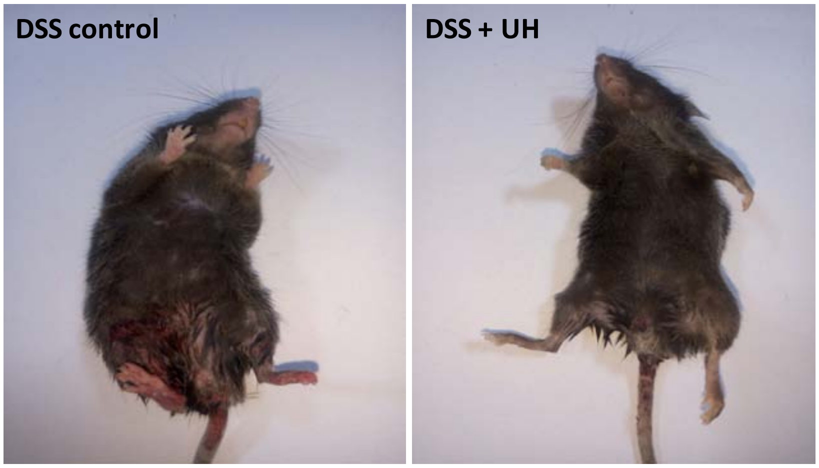


**Supplementary figure S2.** Representative photo of comparison of stool sample from each group at day 12th

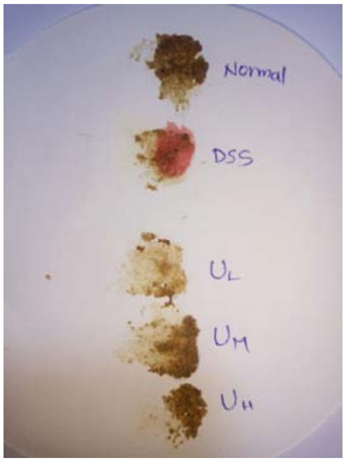

**Supplementary figure S3:** Change in body weight in gm vs time plot

Data presented indicate the mean±SEM (n=6). +++p<0.001 vs. Normal, *p<0.05, **p<0.01 and ***p<0.001 vs. DSS control

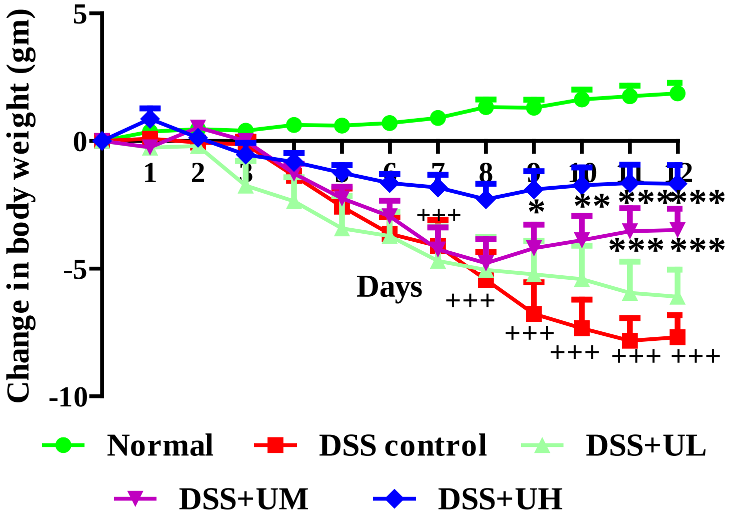

**Supplementary figure S4:** Representative flow diagrams of cytokine panel (IL-6, IL-1β and TNF) determination using cytometric bead array (CBA) assay.

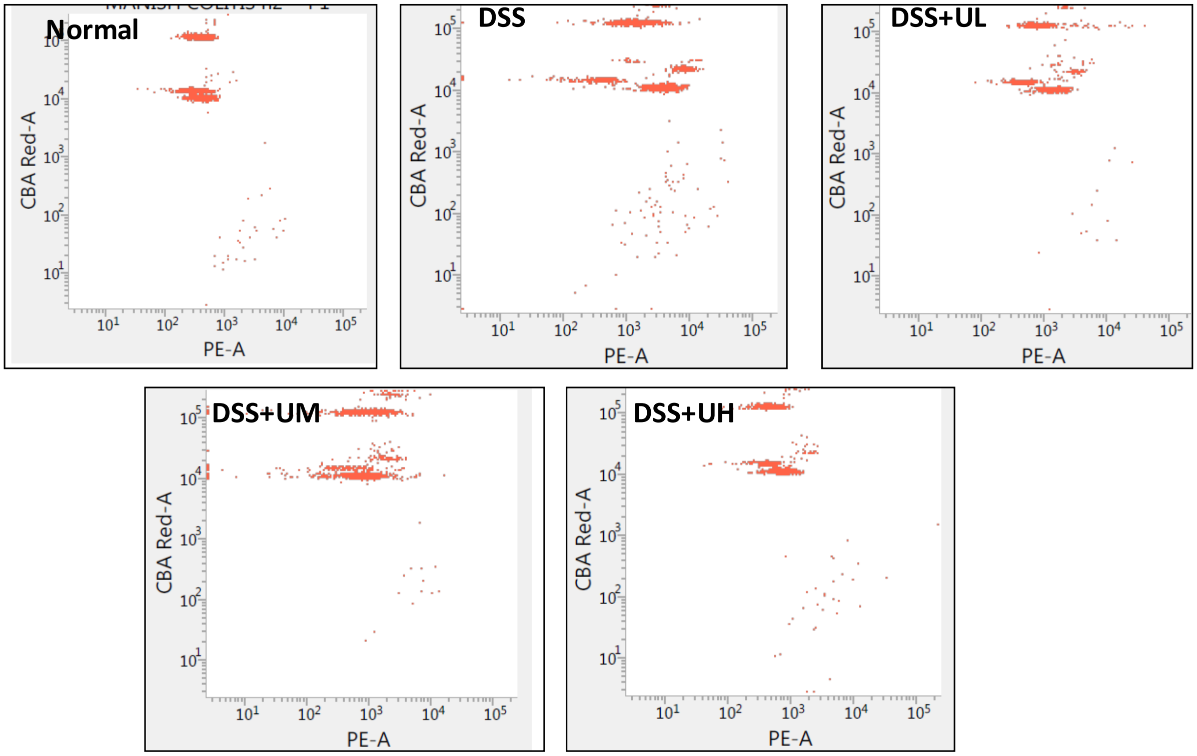

Supplement: Supplementary file 1 — supplementary figures [file 41598_2017_4041_MOESM1_ESM.pdf]
